# Profile of molecular mutations in *pfdhfr*, *pfdhps*, *pfmdr1*, and *pfcrt* genes of *Plasmodium falciparum* related to resistance to different anti-malarial drugs in the Bata District (Equatorial Guinea)

**DOI:** 10.1186/s12936-016-1672-0

**Published:** 2017-01-13

**Authors:** Pedro Berzosa, Andrés Esteban-Cantos, Luz García, Vicenta González, Marisa Navarro, Taiomara Fernández, María Romay-Barja, Zaida Herrador, José Miguel Rubio, Policarpo Ncogo, María Santana-Morales, Basilio Valladares, Matilde Riloha, Agustín Benito

**Affiliations:** 1 Malaria Laboratory, National Centre of Tropical Medicine, Institute of Health Carlos III, C/Monforte de Lemos 5, 28029 Madrid, Spain; 2 Network Collaborative Research in Tropical Diseases, RICET, Madrid, Spain; 3 National Centre of Microbiology, Institute of Health Carlos III, Madrid, Spain; 4 Ministry of Health and Social Welfare of Equatorial Guinea, Malabo, Equatorial Guinea; 5 Instituto Universitario de Enfermedades Tropicales y Salud Pública de Canarias, Universidad de la Laguna, Tenerife, Spain

**Keywords:** Equatorial Guinea, Malaria, *P. falciparum*, Resistance, Mutations, Antimalarial drugs

## Abstract

**Background:**

The emergence of drug resistance in *Plasmodium falciparum* has been a major contributor to the global burden of malaria. Drug resistance complicates treatment, and it is one of the most important problems in malaria control. This study assessed the level of mutations in *P. falciparum* genes, *pfdhfr*, *pfdhps*, *pfmdr1*, and *pfcrt*, related to resistance to different anti-malarial drugs, in the Continental Region of Equatorial Guinea, after 8 years of implementing artesunate combination therapies as the first-line treatment.

**Results:**

A triple mutant of *pfdhfr* (51I/59R/108N), which conferred resistance to sulfadoxine/pyrimethamine (SP), was found in 78% of samples from rural settings; its frequency was significantly different between urban and rural settings (p = 0.007). The 164L mutation was detected for the first time in this area, in rural settings (1.4%). We also identified three classes of previously described mutants and their frequencies: the partially resistant (*pfdhfr* 51I/59R/108N + *pfdhps* 437G), found at 54% (95% CI 47.75–60.25); the fully resistant (*pfdhfr* 51I/59R/108N + *pfdhps* 437G/540E), found at 28% (95% CI 7.07–14.93); and the super resistant (*pfdhfr* 51I/59R/108N + *pfdhps* 437G/540E/581G), found at 6% (95% CI 0.48–4.32). A double mutation in *pfmdr1* (86Y + 1246Y) was detected at 2% (95% CI 0.24–3.76) frequency, distributed in both urban and rural samples. A combination of single mutations in the *pfmdr1* and *pfcrt* genes (86Y + 76T), which was related to resistance to chloroquine and amodiaquine, was detected in 22% (95% CI 16.8–27.2) of samples from the area.

**Conclusions:**

The high level of mutations detected in *P. falciparum* genes related to SP resistance could be linked to the unsuccessful withdrawal of SP treatment in this area. Drug resistance can reduce the efficacy of intermittent prophylactic treatment with SP for children under 5 years old and for pregnant women. Although a high number of mutations was detected, the efficacy of the first-line treatment, artemisinin/amodiaquine, was not affected. To avoid increases in the numbers, occurrence, and spread of mutations, and to protect the population, the Ministry of Health should ensure that health centres and hospitals are supplied with appropriate first-line treatments for malaria.

## Background

**Fig. 1:**
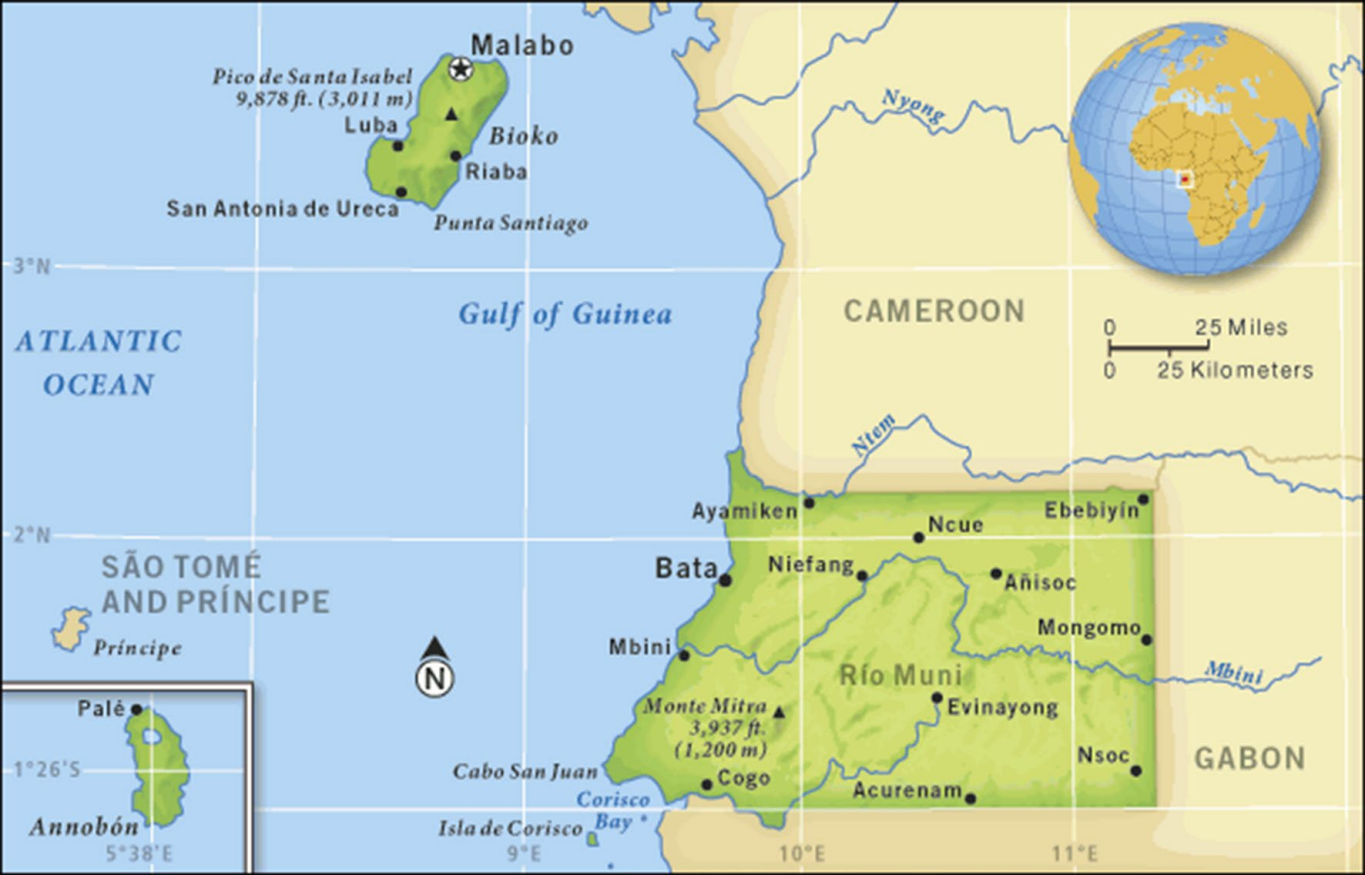
Map of Equatorial Guinea. The map shows the Insular Region, where is located the capital of the country (Malabo); and the Continental Region between Cameroon and Gabon. Source http://go.grolier.com/atlas?id=mgaf016a/72/62272-004-F1EF86B4.jpg

Equatorial Guinea is located in Central West Africa. The country has two regions, the Continental Region (Rio Muni) and the Insular Region (Bioko, Annobon) (Fig. 1). Malaria remains a major public health problem in the country. It is a holo-endemic area with a year-round transmission pattern [1]. The prevalence of malaria was more than 1 case per 1000 people in the population in 2013; the last data published reported 13,000 malaria cases and 66 malaria-related deaths. Malaria in Equatorial Guinea caused 15% of deaths among children under 5 years of age in 2013. In 2014, positive malaria samples reached a frequency of 36% [2].

In 2011, in the continental region, 95.2% of malaria cases were caused by *Plasmodium falciparum* and 9.5% were caused by *Plasmodium vivax.* Moreover, eight cases were caused by mixed infections of *P. falciparum* and *P. vivax* [3]. According to the most recent World Health Organization (WHO) Malaria Report, the prevalence of malaria was 36% in Equatorial Guinea [2].

The emergence of drug resistance, particularly among *P. falciparum* parasites, has been a major contributor to the global burden of malaria in the past three decades [4]. Resistance is the most likely explanation for the doubling of malaria-attributable child mortality in eastern and southern Africa [5]. In general terms, *P. falciparum* drug resistance has become widespread around the world, and this fact makes its treatment difficult. Moreover, *P. falciparum* drug resistance is one of the most important problems in malaria control, due to the increasing resistance to almost all anti-malarial drugs, including amodiaquine (AQ), chloroquine (CQ), mefloquine, artemether–lumefantrine, sulfadoxine/pyrimethamine (SP), and recently, artemisinin. Molecular markers that can detect anti-malarial drug resistance comprise one of the most valuable methods in screening for anti-malarial drugs. Markers can predict the efficacy and resistance to anti-malarial drugs, and indicate emerging resistance in a determined area [6].

The resistance to different anti-malarial drugs is due to single nucleotide polymorphisms (SNPs) in different *P. falciparum* genes, including *pfdhfr, pfdhps, pfcrt*, and *pfmdr1*. The accumulation of SNPs in these parasites can produce in vivo resistance.

It has been demonstrated that the accumulation of SNPs in *pfdhfr* and *pfdhps* genes increases the levels of SP resistance in vivo [7]. In West and Central Africa, a triple mutant genotype of *pfdhfr* (N51I, C59R, S108N) combined with the A437G mutation in the *pfdhps* gene has been related to SP treatment failure [8]. Another significant predictor of SP treatment failure is the quintuple mutant genotype, which includes the *pfdhfr* triple mutant combined with the *pfdhps* double mutant (A437G + K540E) [9].

New terms have been recently introduced to classify SP-resistant parasites. These terms distinguish parasites that are “partially resistant”, “fully resistant”, and “super resistant”. The parasites are classified based on the combination of mutations they carry in the two genes, *pfdhfr* and *pfdhps*. In particular, the combination of triple mutant, *pfdhfr* N51I, C59R, S108N and *pfdhps* A437G, confers partial resistance; the combination of triple mutant, *pfdhfr* N51I, C59R, S108N and double mutant, *pfdhps* A437G, K540E, confers full resistance; and the combination of triple mutant, *pfdhfr* N51I, C59R, S108N and triple mutant, *pfdhps* A437G, K540E, A581G, confers super resistance [10]. These different combinations of mutations, and therefore the three different genotypes, can affect the results of intermittent prophylactic treatment (IPT) in infected pregnant women and children. Moreover, SNPs in *pfmdr1* (the *P. falciparum* multi-drug resistance gene) at positions N86Y and D1246Y were associated with modulating parasite tolerance and susceptibility to a number of anti-malarial drugs, including quinine, AQ, CQ (but here, it plays a secondary role), mefloquine, and lumefantrine [11]. Furthermore, amplifications of the *pfmdr1* gene may cause resistance to artesunate.

Mutations in the *pfcrt* gene at codons 72, 74, 75, and 76 were associated with *P. falciparum* resistance to CQ. Moreover, the K76T mutation was associated with AQ resistance. There is evidence that AQ may induce selection of the *pfcrt* T76 and *pfmdr1* Y86 mutant alleles. That result may provide some insight into the previously observed cross-resistance between CQ and AQ in vivo. Mutant *pfcrt* T76 and *pfmdr1* Y86 alleles are currently used as molecular markers of CQ resistance. They may also be useful for monitoring the spread of AQ resistance in areas of low AQ resistance, such as west Africa [11].

Several studies have investigated molecular markers related to resistance to different anti-malarial drugs in Equatorial Guinea. The most recent studies focused on the detection of mutations in *pfmdr1* and *pfcrt* prevalent in Bioko Island [12, 13]. In Bioko Island, the Ministry of Health has introduced the use of SP as an IPT (IPT-SP), but currently, IPT-SP is not extensively used in the mainland. In the new National Plan against Malaria of Equatorial Guinea, the health authorities plan to introduce an IPT-SP approach in the mainland. Therefore, it is necessary to examine the current prevalence of mutations in *P. falciparum* genes related to SP resistance in this area, to enable assessments of the level of success or failure after implementation.

The *P. falciparum* samples used in this study were collected from the mainland of Equatorial Guinea. They were collected in two previous studies conducted in this region; one was a survey of clinical knowledge and skills regarding malaria; the other was a study on the prevalence of malaria [4, 14]. The present study aimed to investigate the level of mutations related to resistance to different anti-malarial drugs in the *P. falciparum* genes, *pfdhfr*, *pfdhps*, *pfmdr1*, and *pfcrt*, in the Continental Region of Equatorial Guinea, after 8 years of implementing artesunate combination therapies as the first-line treatment for malaria (artesunate/AQ).

## Methods

### Area of study and samples

**Fig. 2:**
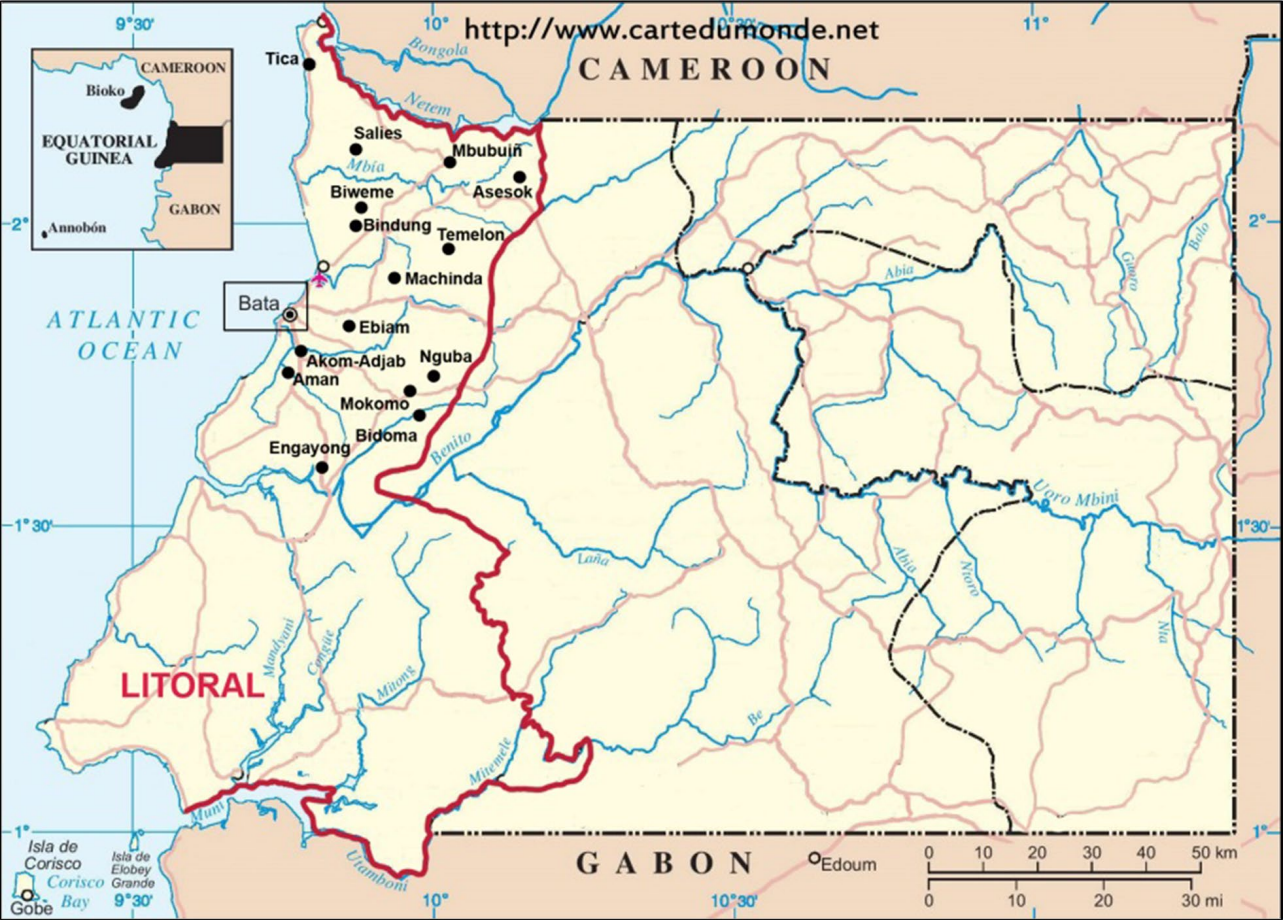
Map of the Mainland of Equatorial Guinea. It appears in *red* the limit of the Litoral Province, whose Capital is Bata. The sampling was carried out in Bata (urban area), and in different rural settings (*black points* in the map). Source https://www.cartedumonde.net, modified

It was carried out a survey in the District of Bata, Continental Region of Equatorial Guinea, located between Cameroon and Gabon, whose capital city is Bata (Fig. 2). This region is divided into four provinces, Centro Sur, Kie-Ntem, Litoral, and Wele-Nzas. It has a tropical climate with two dry seasons (December–March and June–September), alternating with two rainy seasons (March–June and September–December). The mean daily maximum and minimum temperatures range between 29–32 and 19–22 °C, respectively.

Samples were obtained during a cross–sectional survey study, which was carried out in June–August 2013 in the Bata district (Litoral Province), called “PREVAMAL”. That project provided baseline data on malaria prevalence, a molecular characterization of *Plasmodium* and malaria vectors in the area, and information on the knowledge, practices, and attitudes of the general population [4, 14]. Sampling was carried out with a multistage, stratified cluster strategy. Rural villages and urban neighbourhoods were randomly selected with a probability proportional to their size to improve accuracy in sample design. A total of 1043 and 698 people living in urban and rural settings, respectively, were recruited for PREVAMAL study [4] (Fig. 2).

Finger blood samples were taken, and malaria was diagnosed with rapid malaria tests and microscopy. Moreover, blood samples were spotted on Whatman 903™ blood samples were spotted on Whatman 903™ paper (GE Healthcare Bio-Sciences Corp.) for further molecular studies. For example, diagnoses were validated with semi-nested multiplex PCR (to quality control the microscopy and rapid diagnoses; and mutations were analysed in different *P. falciparum* genes that might be related to resistances to different anti-malarial drugs. For the present study, 244 samples (102 from the urban area and 142 from the rural area) were selected to analyse mutations in different *P. falciparum* genes that were related to anti-malarial drug resistance.

### DNA extraction and molecular analysis

DNA was extracted from blood samples (spotted on filter papers) with commercial kits (Speedtools tissue DNA Extraction Kit, Biotools, Spain). Each diagnosis was carried out with the semi-nested multiplex PCR method, as described previously [15, 16].

Samples that were positive for malaria in the semi-nested multiplex PCR, due to *P. falciparum*, were selected to screen for mutations related to drug resistance in the following *P. falciparum* genes: *pfdhfr, pfdhps, pfmdr1*, and *pfcrt*. Mutation screening was performed as previously described in Maryland University Protocols by Dr. C. Plowe (http://medschool.umaryland.edu/malaria/protocols/), with minor modifications. Briefly, first a nested PCR protocol was performed. Then, PCR products were separated with electrophoresis on a 2% agarose gel, and stained with ethidium bromide. With an ultraviolet transilluminator, were identified the correct genes, based on size. Next, was isolated the bands, and digested with different restriction enzymes to analyse restriction-fragment length polymorphisms (RFLPs). Each mutation point in each of the genes requires a different enzyme (New England Biolabs^R^ Inc.) to know whether or not there is a mutation in that position.

Haplotypes of *pfdhfr* and *pfdhps* genes that exhibited a combination of mutations in both genes were classified previously by Naidoo et al. [10]. The first class was a quadruple mutant considered partially resistant (*pfdhfr* 51I59R/108N + *pfdhps* 437G); the second class was a quintuple mutant considered fully resistant (*pfdhfr* 51I/59R/108N + *pfdhps* 437G/540E); and the third class was a sextuple mutant considered super resistant (*pfdhfr* 51I/59R/108N + *pfdhps* 437G/540E/581G). The haplotypes of the other genes studied comprised one double mutation in a single gene: 86Y/1246Y *pfmdr1*; and a combination of two single mutations in different genes: 86Y *pfmdr1* + 76T *pfcrt*.

### Statistical analysis

The frequencies and 95% confidence interval (CI) were used for categorical variables. Distribution of the SNPs in every gene and their combinations in urban and rural settings were assessed by Chi square test or Fisher’s exact test. The level of statistical significance was set at a value of p ≤ 0.05. Statistical analyses were performed using the software package SPSSv.15.0.

### Ethics

This study was approved by the Minister of Health and Social Welfare of Equatorial Guinea (MINSABS) and the Ethics Committee of the Spanish National Health Institute, Carlos III (CEI PI 22_2013-v3). Written informed consent for participation in the study was obtained from the caregivers interviewed and from the heads of the households.

## Results

### Prevalence of SNPs in *pfdhfr* and *pfdhps* genes

**Fig. 3:**
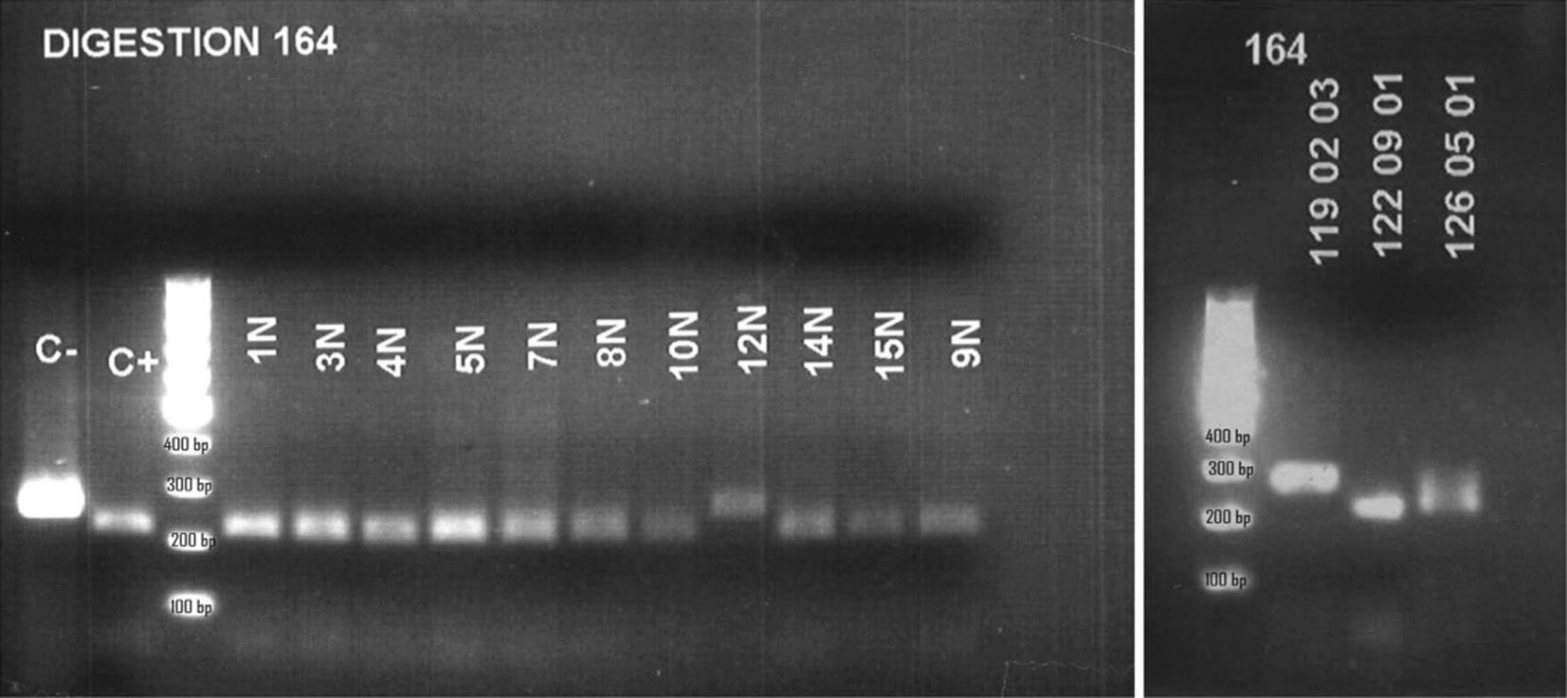
Electrophoresis in agarose gel of the RFLPs for position 164 in *pfdhfr.* Electrophoresis of the result of the digestion with the *PsiI* enzyme. It can be seen that in two samples (12N and 119_02_03) there is no digestion, indicating that the position 164 is mutated (164L) and the enzyme cannot recognize its target. When is digested the fragment of 254 bp, appear two fragments 214 and 42 bp. *C*− Control of “non-digested”, fragment of PCR (size 254 bp) that was not subjected to digestion with *PsiI* enzyme. *C*+ Control of digestion, fragment of the PCR that always is digested with the *PsiI* enzyme

It was examined the individual mutations in each codon of the *pfdhfr* gene. The 51I mutation appeared in 97 and 99% of urban and rural samples, respectively, and the overall prevalence was 98% (95% CI 96.24–97.76). The 108N mutation was found in 99 and 100% of urban and rural samples, respectively, and the overall prevalence was 99% (95% CI 97.75–100.25). Both these mutations had a prevalence close to 100%. The prevalence of the 59R mutation was 72% (95% CI 66.37–77.63). This mutation was found significantly more frequently in rural (79%) than in urban (63%) settings (p = 0.003). The 164L mutation was found in only two samples from rural villages, at a frequency of 1.4% (95% CI −0.25 to 2.25) (Table 1). This was the first report of the detection of this mutation in West Africa. In Fig. 3 appears the result of the RFLPs for the study of the position 164 in *pfdhfr* gene (restriction with the *Psi* I enzyme). When the sample is non-digested, it indicates that has a mutation in 164 position (164L).

**Table 1:** SNPs of each gene by area

Codon	Mutation	Urban area n = 102 N (%)	Rural area n = 142 N (%)	*p* value	Total prevalence n = 244 N (%)	95% CI
*pfdhfr*						
*pfdhfr* N51I	51I	99 (97)	141 (99)	0.174	240 (98)	96.24 to 97.76
*pfdhfr* C59R	59R	64 (63)	113 (79)	*0.003*	177 (72)	66.37 to 77.63
*pfdhfr* S108N	108N	101 (99)	142 (100)	0.209	243 (99)	97.75 to 100.25
*pfdhfr* I164L	164L	0	2 (1.4)	0.337	2 (0.8)	−0.25 to 2.25
*pfdhps*						
*pfdhps* S436A	436A	16 (31)	26 (18)	0.592	42 (17)	12.29 to 21.71
*pfdhps* A437G	437G	94 (92)	126 (89)	0.375	220 (90)	86.24 to 93.76
*pfdhps* K540E	540E	9 (9)	33 (23)	*0.003*	42 (17)	12.29 to 21.71
*pfdhps* A581G	581G	15 (15)	9 (6)	*0.039*	24 (10)	6.24 to 13.76
*pfmdr1*						
*Pfmdr1* N86Y	86Y	68 (67)	104 (73)	0.266	172 (70)	64.25 to 75.75
*Pfmdr1* D1246Y	1246Y	2 (2)	7 (5)	0.224	9 (4)	1.54 to 6.46
*pfcrt*						
*pfcrt* K76T	76T	28 (27)	46 (32)	0.407	74 (30)	24.25 to 35.75

It shows the prevalence of each point of mutation (SNPs) in each gene of *P. falciparum* studied, in urban and rural settings

≤0.05 is taken as significance value

In the *pfdhps* gene, the 437G mutation appeared in close to 90% of samples. Significant differences were found between urban and rural areas in the percentages of samples that harboured the 540E (p = 0.003) and 581G (p = 0.039) mutations. The overall prevalences of these mutations were 17% (95% CI 12.29–21.71) and 10% (95% CI 6.24–13.76), respectively.

The combinations of multiple mutations found in single genes and in both genes, are shown in Table 2. The triple *pfdhfr* mutant (51I/59R/108N, haplotype IRN), which was related to SP resistance in vitro and in vivo, appeared in 62% and 78% of urban and rural samples, respectively (p = 0.007). It was observed another combination that included a mutation (164L), recently detected in this area; this combination, 51I/59R/108N/164L, haplotype IRNL was detected in 1.4% of samples from rural settings. It is important to monitor the spread and increase of this single mutation, to enable the prevention of potential combinations of this mutation with others.

**Table 2:** Combination of mutations related with the resistance in *P. falciparum*

Combination of codons	Combination of amino acids	Number of mutations	Urban area (n = 102) N (%)	Rural area (n = 142) N (%)	*p* value	Total (n = 244) N (%)	95% CI
*pfdhfr*							
51/59/108	IRN	3	63 (62%)	110 (78)	*0.007*	173 (71)	65.31 to 76.69
51/59/108/164	IRNL	4	0	2 (1.4)	0.168	2 (0.8)	−0.32 to 1.92
*pfdhfr* + *pfdhps*							
^a^51/59/108/437	IRNG	4	58 (57)	74 (52)	0.462	132 (54)	47.75 to 60.25
51/59/108/540	IRNE	4	5 (5)	24 (17)	*0.004*	29 (12)	7.92 to 16.08
51/59/108/581	IRNG	4	11 (11)	6 (4)	*0.047*	17 (7)	3.8 to 10.2
51/59/108/164/437	IRNLG	5	0	1 (1)	0.58	1 (0.4)	−0.39 to 1.19
^b^51/59/108/437/540	IRNGE	5	5 (5)	23 (16)	*0.006*	28 (11)	7.07 to 14.93
51/59/108/437/581	IRNGG	5	11 (11)	3 (2)	*0.004*	14 (6)	3.02 to 8.98
^c^51/59/108/437/540/581	IRNGEG	6	2 (2)	4 (3)	0.67	6 (2.4)	0.48 to 4.32
*pfmdr1*							
86/1246	YY	2	1 (1)	3 (2)	0.492	4 (2)	0.24 to 3.76
*pfmdr1* + *pfcrt*							
86/76	YT	2	20 (20)	34 (24)	0.4208	54 (22)	16.8 to 27.2

It shows the prevalence of the different combinations of mutations in each gene (*pfdhfr* and *pfmdr1*), and the combination between different genes of *P. falciparum*: *pfdhfr* ± *pfdhps*

≤0.05 is taken as significance value

^a^Partially resistant *pfdhfr* 51/59/108 + *pfdhps* 437 (51/59/108/437)

^b^Fully resistant *pfdhfr 51/59/108* + *pfdhps* 437/540 (51/59/108/437/540)

^c^Super resistant *pfdhfr 51/59/108* + *pfdhps* 437/540/581 (51/59/108/437/540/437) and the combination in *pfmdr1* ± *pfcrt*

In this study, was detected the partially resistant (51I/59R/108N/437G, haplotype IRNG) at a similar percentage in urban (57%) and rural settings (52%), and the overall prevalence was 54% (95% CI 47.75–60.25). The fully resistant (51I/59R/108N/437G/540E, haplotype IRNGE) appeared in 5 and 16% of urban and rural samples, respectively (p = 0.006). The overall prevalence of the full resistance genotype was 11% (95% CI 7.07–14.93). The super resistant (51I/59R/108N/437G/540E/581G, haplotype IRNGEG) was detected in 2 and 3% of urban and rural samples, respectively, and the overall prevalence was 2.4% (95% CI 0.48–4.32). We also found other combinations with significant differences between urban and rural areas. It was found the quadruple mutant (*pfdhfr* 51I/59R/108N + *pfdhps* 540E; haplotype IRNE) in 5 and 17% of urban and rural samples, respectively (p = 0.004). Another quadruple mutant (*pfdhfr* 51I/59R/108N + *pfdhps* 581G; haplotype IRNG) in 11 and 4% of urban and rural samples, respectively (p = 0.047). It was also found the quintuple mutant (*pfdhfr* 51I/59R/108N + *pfdhps* 437G/581G; haplotype IRNGG) in 11 and 2% of urban and rural samples, respectively (p = 0.004).

### Prevalence of SNPs in *pfmdr1* and *pfcrt* genes

The mutation, *pfmdr1* 86Y, appeared at similar frequencies (67 and 73%) in urban and rural samples, respectively. The overall prevalence of this mutation was 70% (95% CI 64.25–75.75). Another mutation in the *pfmdr1* gene, 1246Y, was less frequently detected than the 86Y mutation (2 and 5% in urban and rural areas, respectively). The overall prevalence of this mutation was 4% (95% CI 1.54–6.46) (Table 1).

The mutation in *pfcrt*, 76T, appeared at a similar frequency in both areas (27% in urban and 32% in rural areas). The overall prevalence of this mutation was 30% (95% CI 24.25–35.75).

The combinations of different mutations in *pfmdr1* and *pfmdr1* + *pfcrt* are summarized in Table 2. The double mutation in *pfmdr1* (86Y + 1246Y; haplotype YY) occurred in 1% and 2% of urban and rural samples, respectively. The overall prevalence was 2% (95% CI 0.24–3.76). Finally, the combination of single mutations in two genes (*pfmdr1* 86Y + and *pfcrt* 76T; haplotype YT), which was related to resistance to CQ and AQ, was found in 20 and 24% of urban and rural samples, respectively; the overall prevalence of this combination was 22% (95% CI 16.8–27.2).

## Discussion

In the current study, a high level of mutations was found in *P. falciparum* genes related to anti-malarial drug resistance in samples from the mainland of Equatorial Guinea. This high prevalence may limit the use of some anti-malarial drugs for treating malaria or for IPTs.

When a country withdraws a given treatment, due to the level of drug resistance, over a given period of time, the sensitive parasite population increases its presence with respect to the resistant population [17]. Furthermore, when a country does not change the treatment policy at the time drug resistance appears, the mutations remain fixed in the population of parasites and the target drugs, like SP, cannot be used, either as treatments or as prophylactics. In the present study, two *P. falciparum* mutations were detected that had a prevalence very close to 100% (108N and 51I). These high prevalences could mean that these mutations are fixed in the parasite population, which implied that SP would have limited effectiveness as an IPT in this area of the country. On the other hand, it has been reported the first detection in West Central Africa of the 164L mutation in *pfdhfr*. This mutation could have important implications for the effectiveness of SP as an IPT, both in children under 5 years old and pregnant women, even though presently, the mutation was detected at a very low frequency. These mutations should be monitored to ensure that the frequency does not increase in the parasite population.

The 581G mutation in *pfdhps* has an important modulatory role in resistance. When the frequency of this mutation is above 10%, IPT with SP cannot protect pregnant women from delivering low birth weight infants [18]. On the other hand, the WHO recommended that, in areas where the frequency of the *pfdhps* 540 mutation is 50%, IPT should not be implemented, because it could fail. In the mainland of Equatorial Guinea, the 581G mutation was detected at 15% in the urban area. For this reason, the National Malaria Control Programme and the Ministry of Health and Social Welfare of Equatorial Guinea should implement measures in the Bata District to control the spread of these mutations. Moreover, it is important to prevent the *pfdhps* 540E mutation from reaching 50% frequency, to avoid reducing the efficiency of IPT-SP.

All three parasite genotypes that confer partial, full, and super resistance [10] were detected in the Bata district. The super resistant genotype has raised the threshold of drug tolerance among parasites, which has important implications for the use of SP. The detection of the super resistant genotype indicates that the SP combination has continued to be used with frequency in this area, despite the fact that the health authorities have withdrawn SP from the national treatment guidelines.

Importantly, it was detected the presence of the 164L mutation in combination with the triple 51/59/108 mutant, although at low frequency (1.4%). It is known that this mutation, alone or in combination with other mutations in *pfdhfr* and *pfdhps*, is related to high SP resistance. Due to the potency of this mutation, an effective control system is required to prevent its spread.

Based on the findings of the high prevalence of mutations in the *pfdhfr* and *pfdhps* genes, it is recommended that the SP combination should not be used as a treatment in Equatorial Guinea. Moreover, any further increases in the mutation levels may require us to reconsider the use of ITP-SP in children under 5 years of age and pregnant women, in this area of the country.

When the prevalence of the super resistant genotype reaches 10%, it is considered sufficiently high to have an effect on the population [10]. In the present study, was found this genotype at frequencies of 2 and 3% in urban and rural samples, respectively. Thus, the prevalence was well below 10%. Nevertheless, the country should put into place measures that can control the rise of this mutation in the population.

In Bioko Island, the Ministry of Health has introduced the use of IPT-SP. Currently, IPT-SP is not extensively used in the mainland; however, the health authorities want to introduce IPT-SP in the mainland region, with the new National Plan Against Malaria of Equatorial Guinea. The presence of these mutant parasites makes it necessary to increase the control over the population at risk, children under 5 years old and pregnant women, that are within the IPT regimen, to avoid a possible reduction in drug efficacy for preventing the disease.

The first time the WHO considered the resistance to SP combination was in 2010 in a technical study. At that time, they recommended that the presence of a 540 mutation in *pfdhps* (one of the SNPs in the quintuple mutant, or fully resistant genotype) should serve as an indicator to predict or to decide where IPT could be established for children under 5 years old [10]. The present study found a high prevalence (16%) of the fully resistant genotype in the rural area.

The 540 mutation in *pfdhfr* is very common in East Africa, where its prevalence was 100% in 2004 [19]. In West Africa, the prevalence has been relatively low; 6.25% in Gabon (2007), 0.8% in Congo (2004), 11% in Sao Tomé, 24% in Nigeria, and 2% in Cameroon [20]. In this study, the prevalence was low in urban samples (9%) but it reached 23% in rural samples. It is important to note that this prevalence was more similar to the prevalence in Nigeria than to the prevalence in Cameroon, which is the neighbouring country. A potential explanation might be that, in Cameroon, from 2009, they adopted a restriction on SP, to be used only in IPT (in pregnant women and children under 5 years old), and they withdrew the use of SP as malaria treatment. In contrast, SP treatment has not been controlled in the area of Equatorial Guinea included in the present study [21].

In areas with a high level of SP resistance, like Northern Tanzania, resistance has been related to the emergence of the super resistant genotype [10]. The presence of this sextuple genotype was shown to be related to the loss of protective efficacy with IPT in children and pregnant women. The frequency of this genotype appeared to increase in cases of placental malaria in women that had received IPT [22]. Currently, the prevalence of the super resistant genotype in the mainland of Equatorial Guinea remains low; but, once again, it is very important to control the use of SP and ensure it is used exclusively for IPT. Furthermore, in the mainland region of Equatorial Guinea, SP should never be used as a treatment, either alone or in combination with another treatment.

The combined *pfdhfr* 51/59/108/164 mutation is common in South America and East Africa, and it has been related to high SP resistance. It is considered fully resistant, when it appears together with the *pfdhps* 437/540 mutation [20]. In the mainland of Equatorial Guinea, this genotype *pfdhfr* 51/59/108/164 was found in only 2 samples from rural settings. The health authorities should be alerted with this finding; increases in these mutations and their spread should be controlled early. The authorities should exhaustively control the withdrawal of SP as a treatment, and limit the use of SP exclusively to IPT implementations in children under 5 years old and pregnant women.

Some resistance genes were detected in Equatorial Guinea at frequencies similar to those reported for Cameroon by Chauvin et al. [20], but other mutations were found for the first time in this part of Africa. In Equatorial Guinea, the *pfdhfr* 51/59/108 genotype was observed less frequently (71%) than in Cameroon (94%), but the partial resistant genotype (*pfdhfr* 51/59/108 + *pfdhps* 437) was the most common. On the other hand, the super resistant genotype (*pfdhfr* 51/59/108 + *pfdhps* 437 + 540 + 581) was not detected in Cameroon, but was detected in Equatorial Guinea, although at low frequency. Importantly, the present study was the first to detect the super resistant genotype in this area of Africa. A previous study conducted in this district [14] found that SP use had been continued, as a treatment alone or in combination with AQ, despite warnings that these treatments should be discontinued to avoid increasing SP resistance. Because SP was not reserved exclusively for IPT, its use may have induced a high level of mutations, which led to the SP resistance detected in this area of Equatorial Guinea.

The mutations found in PF genes *pfmdr1* and *pfcrt* were associated with resistance to other anti-malarial drugs, including artesunate, AQ, lumefantrine. This study was the first to describe these mutations in the mainland of Equatorial Guinea. In 2014 and 2015, Li et al. analysed the *pfmdr1* and *pfcrt* genes in Bioko Island (Equatorial Guinea). They detected an 80% prevalence of the 76 *pfcrt* mutation in Bioko Island [12], but in the Bata district, the prevalence was around 30%. This mutation is related to CQ resistance. This treatment was successfully withdrawn by the authorities in this part of Equatorial Guinea, which could explain the differences in prevalence between this part of the country and Bioko Island or neighbouring countries, like Cameroon (83%) and Gabon (70%) [23, 24].

The combination of the 86Y and 1246Y mutations in the *pfmdr1* gene was associated with reduced susceptibility to artesunate/AQ [6], which is the first-line treatment for uncomplicated malaria in Equatorial Guinea, according to the National Therapeutic Guidelines. It is known that the *pfmdr1* 86Y mutation is related to resistance to CQ and AQ, and the 1246Y mutation is related to quinine resistance [6]. In this study, the prevalence of the combination of both mutations (86Y + 1246Y) was 2% in the parasite population. Based on this relatively low prevalence, currently, the use of artesunate/AQ as a first-line treatment is not endangered in Equatorial Guinea. In studies carried out in Southeast Asia, the presence of mutations in both codons (86 and 1246) has been related to resistance to CQ, mefloquine, and AQ [25, 26]. Mutations in *pfmdr1* were also associated with an increase in resistance to artesunate; therefore, it is estimated that control of these mutations will serve to monitor the resistance to artesunate in a given region [26].

The combination of mutations in *pfcrt* and *pfmdr1* (*pfcrt* 76T + *pfmdr1* 86Y) found in the Bata district are related to AQ resistance. The most recent studies on therapeutic efficacy carried out in the country suggested that artesunate/AQ retained nearly 95% of an effect (unpublished data); thus, currently, the presence of these mutations may not compromise the effectiveness of treatment. However, it becomes necessary to introduce the study of all these mutations in the National Malaria Control Programme taking in account the surveillance of mutations and the spread of them. The present study provided an update on the prevalence of mutations that confer resistance to different anti-malarial drugs (SP, AQ, CQ, mefloquine, artesunate) in the mainland of Equatorial Guinea. Although this study was carried out only in the mainland, our findings indicated that the prevalence of SNPs was different than those reported for Bioko Island and neighbouring countries, such as Cameroon and Gabon. A new mutation (164L) was detected in two samples. The presence of this mutation appeared to be a local phenomenon because, at the time the samples were collected, patients were excluded from the study if they had been in another endemic country in the month prior to taking the sample. Also, the 540E mutation was previously only found in isolates from eastern Africa, and in 2015, it was found in Cameroon [20]. Finally, it was detected the three genotypes described by Naidoo and Roper (partially, fully, and super resistant genotypes). These findings call for continued efforts to prevent the spread of highly drug resistant parasites.

## Conclusions

This study showed that this area had high levels of mutations in the *P. falciparum* genes, *pfdhfr* and *pfdhps*, which were related to resistance to SP treatment. The 164L mutation, which was associated with high resistance to SP, was detected for the first time in this area.

The high number of mutations detected in this area of the country could be linked to the unsuccessful withdrawal of the SP treatment, used alone or in combination with other anti-malarial drugs. This unsuccessful withdrawal could affect the efficacy of IPT for children under 5 years old and for pregnant women. It was also observed mutations in *pfmdr1* and *pfcrt*, which were related to resistance to AQ, CQ, and artesunate. However, a review of the studies on therapeutic effectiveness carried out in this country indicated that these mutations did not affect the effectiveness of first-line treatment with artesunate/AQ.

These results demonstrated the urgency of the necessity to block the use of SP as treatment in this area of the country. The SP combination should be reserved exclusively for use in IPT for children under 5 years old and pregnant women. To avoid the use of drugs prohibited by the public health authorities, it will be necessary to provide the health centres and hospitals with an alternative first-line treatment for malaria, and extend its use to the entire the region. This strategy will limit increases in the number of mutations, the occurrence of new mutations, and particularly, the spread of mutations, to protect the population more effectively.
